# Construction of a Quantitative Structure Activity Relationship (QSAR) Model to Predict the Absorption of Cephalosporins in Zebrafish for Toxicity Study

**DOI:** 10.3389/fphar.2019.00031

**Published:** 2019-01-29

**Authors:** Ying Liu, Xia Zhang, Jingpu Zhang, Changqin Hu

**Affiliations:** ^1^National Institutes for Food and Drug Control, Beijing, China; ^2^Institute of Medicinal Biotechnology, Peking Union Medical College, Chinese Academy of Medical Sciences, Beijing, China

**Keywords:** cephalosporin, QSAR, absorption, liquid chromatography/tandem mass spectrometry, zebrafish, toxicity

## Abstract

Cephalosporins are beta-lactam antibiotics that are widely used in China. Five generations of cephalosporins have been introduced in clinical practice to date; moreover, some new candidates are also undergoing clinical evaluations. To improve the success rates of new drug development, we need to have a comprehensive understanding about the relationship between the structure of cephalosporins and the toxicity that it induces at an early stage. In the cephalosporins toxicity study using zebrafish, the drug absorption is a key point. In this study, we determined the absorption of cephalosporins in zebrafish during toxicity test. The internal concentrations of 19 cephalosporins in zebrafish were determined using a developed liquid chromatography-tandem mass spectrometry (LC-MS/MS) method. Furthermore, a quantitative structure-activity relationship (QSAR) model was established by multilinear regression; moreover, it was used to predict the absorption of cephalosporins in zebrafish. During leave-one-out cross-validation, a satisfactory performance was obtained with a predictive ability (*q*^2^) of 0.839. The prediction ability of the model was further confirmed when the predictive ability (*q*^2^) was 0.859 in external prediction. The best QSAR model, which was based on five molecular descriptors, exhibited a promising predictive performance and robustness. In experiments involving drug toxicity, the developed QSAR model was used to estimate internal concentrations of cephalosporins. Thus, the toxicity results were correlated with the internal concentration of the drug within the larvae. The developed model served as a new powerful tool in zebrafish toxicity tests.

## Introduction

Cephalosporins are one kind of potentβ-lactam antibiotics. A cephalosporin was synthesized for the first time in 1964. Thereafter, five generations of cephalosporins have been developed for clinical use (Bryskier, 2000; Hwu et al., 2003; Singh, 2004; Butler and Cooper, 2011). Cephalosporins were derived from the core structure of 7-aminocephalosporanic acid (7-ACA), which attracted a lot of attention from chemists. The molecule 7-ACA had a unique structure, which could be modified to develop new antibiotic candidates; however, these new antibiotic candidates have to be clinically evaluated before being introduced into the market (Macheboeuf et al., 2007). To minimize the attrition rates of drug development, the toxicity of candidate drugs must be determined accurately. This is a crucial part of the drug discovery process. Drug toxicity is a chief reason for withdrawing drugs from the market (Li, 2001, 2004). Thus, toxicity assessment in the early stage of drug discovery can increase the chances that a drug will reach consumers. Currently, the success rate of new drug development is very low. To improve this parameter, we need to have a comprehensive understanding of the relationship between toxicity and drug structure.

In previous studies, the toxic effects of cephalosporins were determined by zebrafish embryo toxicity testing (Zhang et al., 2010, 2013, 2015; Chen et al., 2017; Qian et al., 2018). One of a preliminary conclusion was that the toxic effects of drugs were both influenced by toxic functional groups and drug absorption. The procedure for zebrafish embryo toxicity testing is as follows: zebrafish was typically administered with aqueous solutions of compounds. The diffusion of these compounds predominantly occurred through the skin (Ali et al., 2012; Padilla et al., 2012; Tal et al., 2017). Drug uptake is governed by the physicochemical properties of each compound. The aqueous concentration of the drug in the exposure medium is not necessarily equal to the concentration of the drug in the tissue of zebrafish embryo/larvae (Padilla et al., 2012). In fact, some investigators have pointed out that there is a discrepancy between aqueous concentration and the body burden of chemicals in zebrafish embryo/larva; however, this discrepancy has been observed for only select chemicals (Padilla et al., 2012). To evaluate drug toxicity, it is necessary to determine the internal concentration of drugs in zebrafish.

The quantitative structure–activity relationship (QSAR) model is one of the most popular computer-aided tools, which are employed in medicinal chemistry for drug discovery (Wang et al., 2015). Using QSAR model, a combination of theoretical or semi-experimental calculations and statistical analysis were performed to determine the relationship between molecular structure descriptors and physicochemical properties or biological activities of compounds. The methods were widely used for predicting various physicochemical properties and biological activities of organic compounds; these compounds have wide applications in pharmaceutical industry (Chen, 2015; Thareja, 2015; Raczakgutknecht et al., 2017; Lo et al., 2018). One of the primary application areas is helping researchers understand and exploit the relationship between chemical structures and their biological activities. In general, a suitable set of chemical descriptors is generated from a series of active and inactive molecules. A model is then constructed to determine the relationship between chemical descriptors and the bioactivity of interest. The developed model is then rigorously validated to select those that show best performance. Finally, the model is used to predict the activity of test compounds (Wang et al., 2015). The developed model can accurately predict *in silico* how chemical modifications might influence biological behavior. Many physiochemical properties of drugs, such as toxicity, metabolism, drug–drug interactions, and carcinogenesis, have been effectively determined by QSAR techniques (Cherkasov et al., 2014). In previous studies, QSAR models involved the use of different molecular descriptors and statistical methods. These models were used to determine the absorption of drugs in human intestine (Shen et al., 2014; Basant et al., 2016; Edwards et al., 2016; Liu, 2018).

To the best of our knowledge, there is no QSAR model for predicting the absorption of cephalosporins in zebrafish. Using the developed LC-MS/MS method (Liu et al., 2018), we determined the absorption of 19 cephalosporins in zebrafish. Then, a QSAR model was developed using the absorption data we obtained. The QSAR model was used to predict the absorption of other cephalosporins, which were used in the toxicity test in zebrafish.

## Materials and Methods

### Chemicals and Reagents

Cefalexin, cefradine, cefadroxil, cefaclor, ceftizoxime, cefuroxime, cefoxitin, ceftezole, cefazolin, cefotaxime, cefathiamidine, flomoxef, cefmenoxime, cefpirome, cefminox, cefotiam, ceftazidime, cefodizime, cefoperazone, and clenbuterol [internal standard (IS)] were national reference substances, which were obtained from the National Institutes for Food and Drug Control (Beijing, China) (Table 1). Ammonium hydroxide and acetonitrile were purchased from Fisher Scientific International (Fair Lawn, NJ, United States). Formic acid was purchased from Sigma-Aldrich Corporation (St. Louis, MO, United States). A stock solution (1.0 mg/mL) of each of the cephalosporins was prepared by dissolving each reference standard in deionized water. This stock solution was stored at -70°C. A stock solution of ammonium formate buffer was prepared by mixing ammonium formate (1.0 M) and formic acid (1.0 M). The resultant stock solution was stored at 4°C. Different cephalosporins were dissolved in breeding water, which is artificial sea water prepared by dissolving instant ocean sea salt (CNSG, Tianjin, China) in deionized water, respectively.

**Table 1:** Molecular formula and MRM parameters of each analyte.

Drug	Molecular formula	Precursor ion (m/z)	Product ion (m/z)	DP (V)	CE (eV)	CXP (V)
Cefalexin	C_16_H_17_N_3_O_4_S	348	106	40	34	10
Cefradine	C_16_H_19_N_3_O_4_S	350	176	45	40	11
Cefadroxil	C_16_H_17_N_3_O_5_S	364	114	35	40	9
Cefaclor	C_15_H_14_ClN_3_O_4_S	368	106	50	27	11
Ceftizoxime	C_13_H_13_N_5_O_5_S_2_	384	126	48	45	10
Cefuroxime	C_16_H_16_N_4_O_8_S	442	364	21	20	9
Cefoxitin	C_16_H_17_N_3_O_7_S_2_	445	215	26	30	10
Ceftezole	C_13_H_12_N_8_O_4_S_3_	441	156	46	26	8
Cefazolin	C_14_H_14_N_8_O_4_S_3_	455	156	48	25	13
Cefotaxime	C_16_H_17_N_5_O_7_S_2_	456	167	65	27	9
Cefathiamidine	C_19_H_28_N_4_O_6_S_2_	473	159	61	50	12
Flomoxef	C_15_H_18_F_2_N_6_O_7_S_2_	497	122	50	61	10
Cefmenoxime	C_16_H_17_N_9_O_5_S_3_	512	324	45	28	11
Cefpirome	C_22_H_22_N_6_O_5_S_2_	515	396	50	15	5
Cefminox	C_16_H_21_N_7_O_7_S_3_	520	161	65	28	9
Cefotiam	C_18_H_23_N_9_O_4_S_3_	526	174	61	50	10
Ceftazidime	C_22_H_22_N_6_O_7_S_2_	547	167	45	40	10
Cefodizime	C_20_H_20_N_6_O_7_S_4_	585	125	40	75	11
Cefoperazone	C_25_H_27_N_9_O_8_S_2_	646	290	57	36	10
Clenbuterol (IS)	C_12_H_18_Cl_2_N_2_O	277	203	35	23	8


### Zebrafish Embryo Treatment by Cephalosporins

Zebrafish (*Danio rerio*) were fed at the Institute of Medicinal Biotechnology, Beijing Union Medical College, Beijing, China. In a study conducted by Zhang et al. (2013), the breeding water was prepared for zebrafish with instant ocean sea salt (CNSG, Tianjin, China). Zebrafish wild-type (WT) embryos (*n* = 30) at 6 h post-fertilization (6 hpf) were used. After immersing the embryos in 2 mL of each drug solution (Drugs were dissolved in breeding water), they were incubated in a 20 mm dish for 3 days. Three concentrations of each drug (0.28, 1.0, and 2.0 mM) were used for incubating 6 hpf zebrafish embryos, and each experiment was repeated at least thrice. The zebrafish bodies were collected at 3 days post-fertilization (dpf). The blank controls were zebrafish WT embryos (*n* = 30) that were incubated in breeding water.

All experimental protocols were approved by Institute of Medicinal Biotechnology, Beijing Union Medical College, Beijing, China. These experiments were performed in accordance with the Good Laboratory Practice Regulations for Non-clinical Laboratory Studies of Drugs, which was issued by the National Scientific and Technological Committee of the People’s Republic of China.

### Determination of Drug Absorption in Zebrafish by LC-MS/MS Method

The anesthetic MS-222 (3-aminobenzoic acid ethylester, methanesulfonate salt, Sigma) was added to the larval solution until its final concentration was 0.016%. Using a cell strainer (100 μm, BD-Falcon, United States), zebrafish bodies (30 in each dish) were washed five times with deionized water. Zebrafish bodies were triturated in 100 μL of deionized water, and then they were homogenized for 1 min. Zebrafish homogenate (50 μL) and internal standard (IS) working solution (10 μL) were individually transferred into a 1.5-mL microcentrifuge tube. Then, 200 μL of 1% formic acid-methanol solution was added into the tube. The samples were vortexed for 20 s. To precipitate proteins, the samples were centrifuged at 10,800 *g* for 10 min. The resultant supernatant (10 μL) was injected into LC-MS/MS for analysis.

### LC-MS/MS Method

The LC-MS/MS analysis was carried out by using the validated method, which was developed by our group (Liu et al., 2018). The LC-MS/MS technique was performed by combining a 20A LC (Shimadzu, Kyoto, Japan) and a 6500 Q-Trap mass spectrometer (AB Sciex, Foster City, CA, United States), which was equipped with an electrospray ionization (ESI) source. Moreover, LC separations were performed on a reversed-phase ACE C_18_ column (5 cm × 2.1 mm ID, 3 μm particle size, Advanced Chromatography Technologies, Aberdeen, United Kingdom), which was attached to a corresponding guard column (1 cm × 2.1 mm ID, 3 μm particle size; Advanced Chromatography Technologies, United Kingdom). The mobile phase comprised of 5 mM ammonium formate (pH = 3.5) (solvent A) and acetonitrile (solvent B). The composition of the gradient was as follows: 2% solvent B for the first 0.1 min; 3.5 min, 90% solvent B; 6.0 min, 90% solvent B; 6.1 min, 2% solvent B; and 10 min, 2% solvent B. Mass spectrometric analysis was performed in a positive mode. Ion-spray voltage was set at 5000 V, and source temperature was kept fixed at 500°C. Curtain gas, gas 1, and gas 2 were 25, 40, and 40 psi, respectively. Multiple reaction monitoring (MRM) scan was used for the quantification of 19 analytes. In this scan, we determined the most intense product ion (PI) of each analyte. Table 1 presents the compound-dependent parameters of each analyte. The entrance potential (EP) of all analytes was 10 eV. For each ion transition, dwell time and pause time were set to 30 and 5 ms, respectively. The Analyst 1.6.2 software (AB Sciex, Foster City, CA, United States) was used to perform data acquisition and analysis.

### Data Set and Descriptor Generation

Cephalosporins are currently grouped into four generations. The widely used cephalosporins in market are mainly chemicals with ammonia thioxime group at C-7, chemicals with tetrazole group at C-3 and oral cephalosporins with simple substituents such as methyl group. The detailed development history of cephalosporins can be found in the following paper (Bryskier, 2000). Although only 19 analogs were selected in the model, they can totally represent all the cephalosporins widely used in the clinical. The 3D chemical structures of all drugs were obtained from PubChem Compound database. Before minimizing energy, we defined the existing forms of all drugs in the zebrafish. The predicted pKa values of each drug were determined by using Discovery Studio 4.0 software (Accelrys Software Inc., San Diego, CA, United States). We had to determine the molecular conformation of lowest energy prior to computing relevant parameters. For this purpose, structures were energy-minimized by using DS 4.0 software. For energy minimization, the smart minimizer algorithm was implemented by using Chemistry at Harvard Macromolecular Mechanics (CHARMM) force field. The Smart Minimizer algorithm performed 5000 maximum steps with a residual mean square (RMS) gradient, which has a tolerance of 0.01 kcal/(mol ×Å). Then, we performed conjugate gradient minimization. We used an implicit solvent model termed distance-dependent dielectrics, and the dielectric constant was set to 80. For calculating molecular descriptors, we considered all the minimized compounds in the next step.

A set of 2D and 3D molecular descriptors were calculated by using DS 4.0 software. The software DS 4.0 provided the following molecular descriptors: AlogP series, molecular properties (logD and pKa), surface area and volume, topological descriptors, dipole, jurs descriptors, principal moments of Inertia, and shadow indices. The meaning of all descriptors can be determined with the help of DS 4.0 software. After eliminating small variance, under-represented, and non-numerical variables, we selected 148 molecular descriptors for further QSAR analysis.

### QSAR Study

Dose-internal concentration curves were constructed by linear regression analysis. The slope of each linear regression was defined as *k*-value for each drug. The *k*-values of 19 drugs were considered as output variables in QSAR study. The entire data set was split into the training set and test set by a random index, which was operated by DS4.0 software. The training set percentage was set at 80. To build and train the model, we did not use data points of the test set; however, these data points were used after model building in order to judge the performance of the model by external validation.

Genetic Function Approximation (GFA) was used to further select the key variables and build QSAR models. In some ways, GFA is a model-selection procedure that is analogous to stepwise regression; however, the key difference is that the GFA produces a population of models (e.g., 100), instead of generating a single model [DS]. To determine GFA, we combined multivariate adaptive regression splines (MARS) algorithm with genetic algorithm. Our main objective was to evolve the population of equations that fit best with the training set data. It provides an error measure called the lack-of-fit (LoF) score, which automatically penalizes models with too many features. Splines may also be used as a powerful tool for non-linear modeling. The range of variations in this population would give more information about the quality of fit and the importance of descriptors. Thus, GFA algorithm is a simple and powerful way through which we can obtain an accurate interpolative model, which is a superior model as compared to other similar techniques (Rogers and Hopfinger, 1994; Fan et al., 2001).

The functional form of the model was linear. The obtained model would lose statistical significance with the inclusion of too many descriptors; therefore, the number of descriptors was restricted between 2 to 6 in these models. The key parameters of GFA were as follows: the population size was 100; maximum generations were 5000; maximum correlation threshold for any two variables was 0.95 in the input data; other parameters were considered as default values. Friedman LOF values were used to determine the quality of obtained equations. Finally, we obtained 100 GFA models.

The following statistical parameters of models were calculated by DS 4.0 software: *r*^2^ is the coefficient of determination; *r*^2^_adj_ is *r*^2^ adjusted for the number of terms in the model; *r*^2^_pred_ is the prediction (PRESS) *r*^2^, which is equivalent to *q*^2^ from a leave-1-out cross validation; LoF is the Friedman lack-of-fit score; *p* is the *p*-value for the significance of regression; and RMSE is defined as root mean square error. In addition, an external validation was used to determine the model’s generalization and predictive accuracy. It was measured by squared correlation coefficient (*q*^2^) and root mean square error of external validation (RMSE-EV). The model shows both better model statistics and external validation statistics, which were used for selecting a lower number of input variables.

## Results and Discussion

### Determination of the Absorption of Cephalosporins in Zebrafish Embryo

**FIGURE 1:**
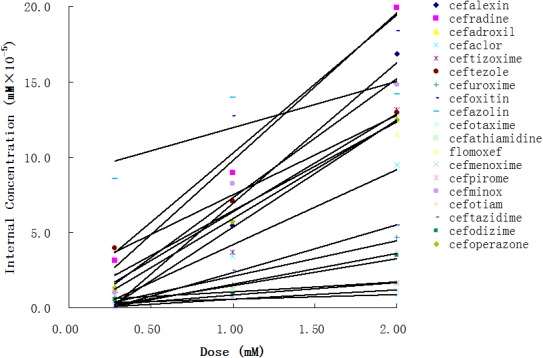
The dose-internal concentration curves were obtained for 19 cephalosporins.

Using the aforementioned method, we analyzed the internal concentrations of cephalosporins in zebrafish. To obtain the calibration curve of each analyte, we prepared calibration samples (1, 5, 10, 25, 50, 75, 100, 250, and 500 ng/ml) of each cephalosporin in blank zebrafish homogenate. The calibration samples and administration samples were processed in a timely manner. The internal concentration of each cephalosporin in the zebrafish was calculated by using the corresponding calibration curve (Table 2). Table 2 shows the results of internal concentrations determined in zebrafish. Zebrafish embryos were exposed to three concentration solutions of each drug (0.28, 1.0, and 2.0 mM) at 6 hpf–3 dpf. There were significant differences between the internal concentrations of different cephalosporins, and indicated that the absorption of each drug was different. Dose-internal concentration curves were constructed by linear regression analysis (Figure 1). For each drug, the slope of each linear regression was defined as *k*-value. This parameter was used to evaluate the absorption capacity of each drug. As shown in Table 3, the *k*-value of each drug was in the range of 0.3018–9.8111. The drug was more readily absorbed when the *k*-value was higher in magnitude. Cefotaxime, cefathiamidine, cefmenoxime, cefpirome, cefodizime, cefotiam, cefuroxime, cefazolin, and ceftazidime were the drugs whose *k*-value was lower (<5). This indicates that these drugs are not easily to be absorbed into the system of zebrafish. Cefalexin, cefradine, cefadroxil, cefaclor, ceftizoxime, ceftezole, cefoxitin, flomoxef, cefminox, and cefoperazone were the drugs whose *k*-value was higher in number (>5). This indicates that these drugs can be absorbed easily.

**Table 2:** Calibration curve and absorption results of zebrafish.

			Internal concentration (mM × 10^-5^)		
			
Drug	Calibration curve	r	0.28 mM	1 mM	2 mM
Cefalexin	*y* = 0.00175*x* + 0.00108	0.9962	1.01	5.48	16.83
Cefradine	*y* = 0.000156*x* + 0.000912	0.9918	3.15	8.93	19.89
Cefadroxil	*y* = 0.000107*x* + 0.000555	0.9967	1.32	7.07	12.55
Cefaclor	*y* = 0.000564*x* + 0.000483	0.9959	1.01	3.43	9.49
Ceftizoxime	*y* = 0.000732*x* + 0.000101	0.9969	1.17	3.70	13.15
Ceftezole	*y* = 0.000141*x* + 0.00122	0.9948	3.95	7.08	12.96
Cefuroxime	*y* = 0.0000936x + 0.00106	0.9950	0.68	1.55	4.66
Cefoxitin	*y* = 0.000205*x* + 0.000846	0.9899	2.22	12.73	18.39
Cefazolin	*y* = 0.000427*x* + 0.000186	0.9969	8.56	13.95	14.19
Cefotaxime	*y* = 0.00175*x* + 0.00108	0.9962	0.27	0.75	0.82
Cefathiamidine	*y* = 0.00562*x* + 0.00164	0.9969	0.49	1.29	1.63
Flomoxef	*y* = 0.0000711*x* + 0.000339	0.9953	1.07	8.30	11.54
Cefmenoxime	*y* = 0.000587*x* + 0.00268	0.9976	0.00	0.71	1.14
Cefpirome	*y* = 0.00509*x* + 0.00295	0.9969	0.21	0.86	1.67
Cefminox	*y* = 0.00146*x* + 0.0042	0.9960	1.01	8.26	14.78
Cefotiam	*y* = 0.0000419*x* + 0.000272	0.9935	0.25	1.31	3.73
Ceftazidime	*y* = 0.000259*x* + 0.000296	0.9981	0.00	2.45	5.47
Cefodizime	*y* = 0.000281*x* + 0.00268	0.9959	0.51	0.94	3.47
Cefoperazone	*y* = 0.000061*x* + 0.00102	0.9986	1.30	5.75	12.47


**Table 3:** The existing form and *k*-value of each drug.

No.	Name	The existing form in zebrafish	*k*-value	No.	Name	The existing form in zebrafish	*k*-value
(1)	Cefathiamidine	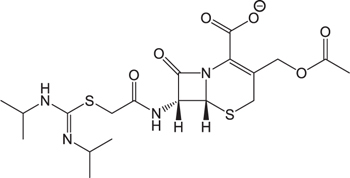	0.6439	(11)	Cefodizime	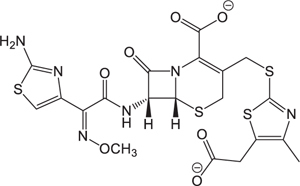	1.7709
(2)	Cefradine	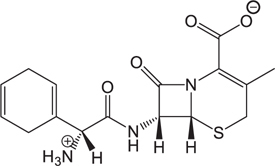	9.8110	(12)	Cefuroxime	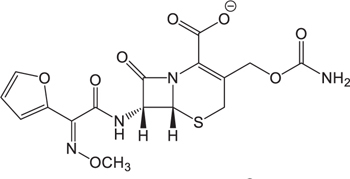	2.3643
(3)	Cefotiam	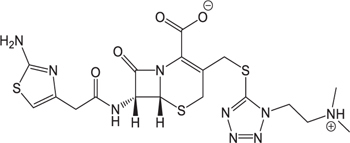	2.0488	(13)	Cefpirome	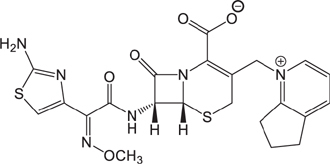	0.1689
(4)	Cefoperazone	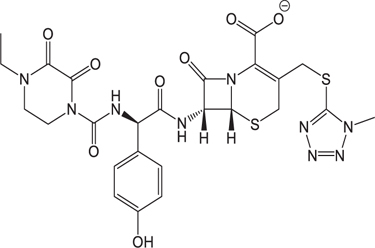	6.5063	(14)	Ceftazidime	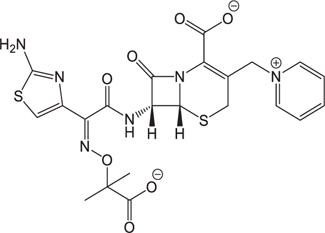	3.1702
(5)	Cefadroxil	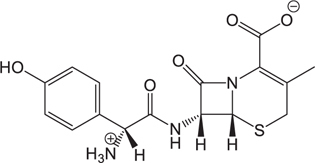	6.4617	(15)	Cefotaxime	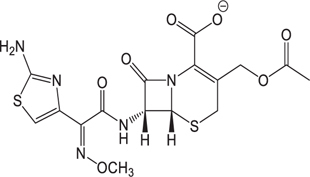	0.3023
(6)	Cefaclor	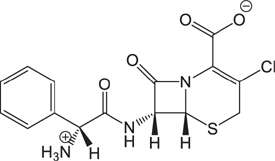	5.0027	(16)	Ceftizoxime	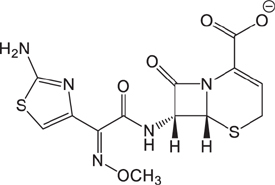	7.1156
(7)	Ceftezole	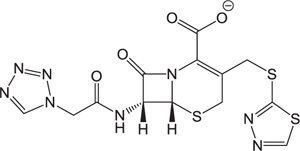	5.2798	(17)	Cefmenoxime	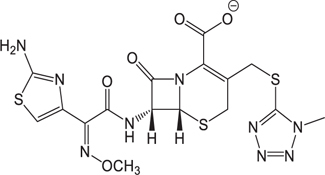	0.6493
(8)	Flomoxef	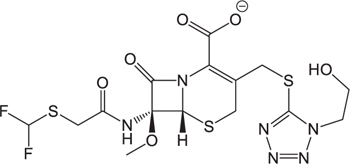	5.9111	(18)	Cefalexin	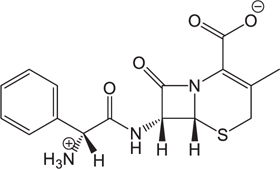	9.3280
(9)	Cefminox	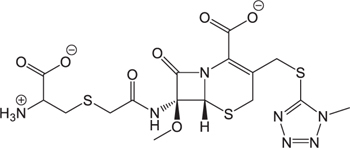	7.9122	(19)	Cefazolin	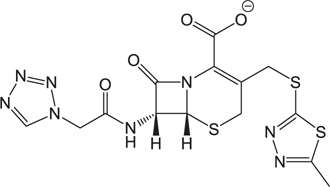	3.0851
(10)	Cefoxitin	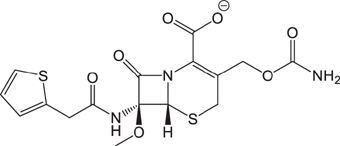	9.1638	/	/	/	/


For approximately 72 h, developing zebrafish embryos are surrounded by the chorion. The zebrafish chorion is a 1.5–2.5 μm thick acellular envelope. The chorion membrane complex to consist of three layers: the middle and inner layers are pierced by cone-shaped pore canals which display a corkscrew-like ridged wall with lamellae ringing the inner surface (Bonsignorio et al., 1996). The chorion of zebrafish has been discussed as a potential barrier for the uptake of chemical substances (Pelka et al., 2017). So in zebrafish embody toxicity testing, the uptake of chemicals is a complex procedure. The absorption of chemicals in zebrafish could not be estimated only based on simple property of the compound, such as polar surface area (PSA) and lipophilicity (logP or logD) (Kislyuk et al., 2017). However, based on the absorption data we determined in zebrafish, we could use QSAR model to predict the absorption of other cephalosporins or impurities in zebrafish. This would enable us to thoroughly compare the uptake and toxicity of the drug in zebrafish.

### Construction of the QSAR Model for Cephalosporins

The pH of zebrafish was about 7.0; therefore, the existing forms of all drugs in the zebrafish could be determined by combining the predicted pKa values. Table 3 enlists the actual existing structures of 19 cephalosporins. In the model development step, 15 cephalosporins were used as training set. The remaining four (Nos. 1, 3, 10, and 16 in Table 1) cephalosporins were used to evaluate the prediction ability of the model. After optimizing the number of descriptors, the best QSAR model was constructed by using five descriptors in the following equation: *k* = -30.23–2.0167 ^∗^ HBD_Count -0.03463 ^∗^ Jurs_WPSA_1 + 1.6392 ^∗^ Num_Aliphatic Single Bonds + 0.72925 ^∗^ Num_Aromatic Bonds + 18.652 ^∗^ QED_MW (1).

*n* = 15, *r*^2^ = 0.928, *r*^2^_adj_ = 0.887, *r*^2^_pred_ = 0.839, RMSE = 1.065, LoF = 5.888, *P* = 7.013e-05; *q*^2^ = 0.859, RMSE-EV = 3.555.

**FIGURE 2:**
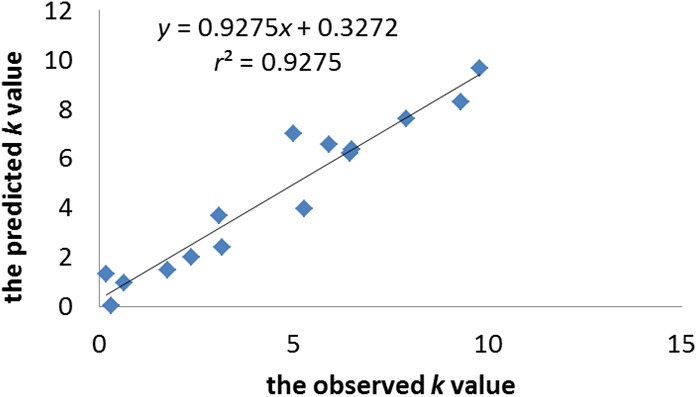
The plot of the observed *k-*value vs. the predicted *k-*value of the training set.

The statistical significance of the model was confirmed by *p*-value, which was smaller than the critical value at 95% confidence limit. The *r*^2^_adj_ value of model indicates that the model can explain 88.7% of the variance, whereas its *q*^2^ value shows the 85.9% predictive variance of the model. The small difference between *r*^2^ and *r*^2^_prep_ (less than 0.1) confirms that the model is not over-fitted. Figure 2 illustrates the plot of the observed *k*-value vs. the predicted *k*-value of the training set. It can be seen that the predicted data of this model is in accordance with the experimental results. The performance of the model is also validated by comparing statistical parameters of the training set with those of the test set. The determination coefficient and RMSE of the training and external test sets were very similar, which confirms that the prediction ability of the model was good for cephalosporins. Moreover, the built model was used to accurately predict and evaluate the absorption ability of cephalosporins in zebrafish without doing complex experiments.

The molecular descriptors involved in model development are as follows: HBD_Count, Jurs_WPSA_1, Num_AliphaticSingleBonds, Num_AromaticBonds, and QED_MW. Table 4 presents the meanings of these molecular descriptors, which are related to hydrogen bond donation, molecular shapes, electronic configuration, and drug-likeness information of compounds. The *p*-values (Table 4) of each molecular descriptor were smaller than the critical value at 95% confidence limit. This indicates that the statistical significance of selected molecular descriptors. Smaller the *p*-value of molecular descriptor, more influential would be the *k*-value of the molecular descriptor. Num_AliphaticSingleBonds and QED_MW are the most important molecular descriptors in the five. Besides, it can be seen from Eq. (1) that Num_AliphaticSingleBonds, Num_AromaticBonds, and QED_MW have positive contribution to the *k*-value of drugs; however, HBD_Count, and Jurs_WPSA_1 have a negative effect on the *k*-value of drugs.

**Table 4:** The meaning and *p*-value of each five molecular descriptors in the final model.

Molecular descriptor	Meaning	*p*-value
HBD_Count	Number of hydrogen bond donating groups in the molecule. It is calculated using substructure queries to count the number of times the queries match structural features in the molecule	2.923e-02
Jurs_WPSA_1	Surface-weighted charged partial surface areas. Set of six descriptors obtained by multiplying descriptors 1–6 by the total molecular solvent-accessible surface area and dividing by 100	2.542e-03
Num_AliphaticSingleBonds	Single bonds in aliphatic systems	1.928e-05
Num_AromaticBonds	Bonds in aromatic ring systems	2.294e-03
QED_MW	Molecular weight contribution to the quantitative estimate of drug-likeness (QED) score	2.054e-05


**FIGURE 3:**
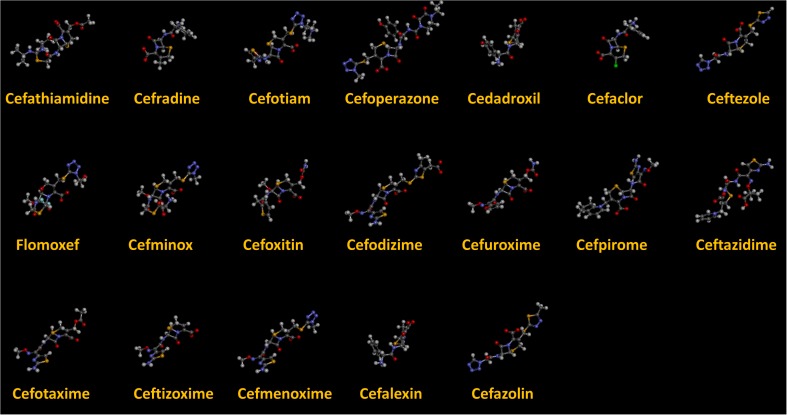
The 3D conformations of the 19 analogs in zebrafish.

As we can see from the model, the descriptors selected are mostly 3D descriptors and they are determined by the 3D structures of the analogs. Though the analogs are very similar in terms of their 2D structures, their 3D structures are totally different (Figure 3). For example, the 2D structures of cefradine and cefaclor are very similar as is shown in Table 3, however, their 3D structures are different. In the zebrafish, the absorption of drugs is mainly determined by the 3D structures, so this maybe the main reason why cephalosporins have different *k*-values in zebrafish even though they all have the core structure of 7-ACA. Differences of substitutes at the periphery of their structures can have an important effect on their 3D conformations and further influence their absorption in zebrafish. This study enlightens the necessity of monitoring the absorption of cephalosporins analogs in zebrafish and also provides a creative way of predicting the absorption via QSAR.

## Conclusion

In this experiment, the internal concentrations of 19 cephalosporins were determined by a developed LC-MS/MS method in zebrafish. An accurate and simple QSAR model was built with five molecular descriptors to predict the absorption of cephalosporins using the obtained internal concentration data of zebrafish. The prediction ability was confirmed by statistical significance and external validation. To the best of our knowledge, this is the first study that predicts the absorption of zebrafish by using the QSAR model. Moreover, this model is used to predict the absorption data of other cephalosporins without involve complex experiments. In addition, this model is also used to predict the absorption data of cephalosporins’ impurities, which have organic structures similar to those of cephalosporins. Thus, the absorption data can be correlated with toxicity results, which illustrate that the actual concentration of the drug within the larvae of zebrafish. This is a powerful tool to determine the early stage of toxicity during new drug development for new antibiotics.

## Author Contributions

All authors listed have made a substantial, direct and intellectual contribution to the work, and approved it for publication.

## Conflict of Interest Statement

The authors declare that the research was conducted in the absence of any commercial or financial relationships that could be construed as a potential conflict of interest.

## References

[B1] Ali S. Champagne D. L. Richardson M. K. (2012). Behavioral profiling of zebrafish embryos exposed to a panel of 60 water-soluble compounds. *Behav. Brain Res.* 228 272–283. 10.1016/j.bbr.2011.11.020 22138507

[B2] Basant N. Gupta S. Singh K. P. (2016). Predicting human intestinal absorption of diverse chemicals using ensemble learning based QSAR modeling approaches. *Comput. Biol. Chem.* 61 178–196. 10.1016/j.compbiolchem.2016.01.005 26881740

[B3] Bonsignorio D. Perego L. Del Giacco L. Cotelli F. (1996). Structure and macromolecular composition of the zebrafish egg chorion. *Zygote* 4 101–108. 10.1017/S0967199400002975 8913023

[B4] Bryskier A. (2000). Cephems: fifty years of continuous research. *J. Antibiot.* 53 1028–1037. 10.7164/antibiotics.53.1028 11132947

[B5] Butler M. S. Cooper M. A. (2011). Antibiotics in the clinical pipeline in 2011. *J. Antibiot.* 64 413–425. 10.1038/ja.2011.44 21587262

[B6] Chen B. (2015). Development of quantitative structure activity relationship (QSAR) model for disinfection byproduct (DBP) research: a review of methods and resources. *J. Hazard. Mater.* 299 260–279. 10.1016/j.jhazmat.2015.06.054 26142156

[B7] Chen B. Gao Z.-Q. Liu Y. Zheng Y.-M. Han Y. Zhang J.-P. (2017). Embryo and developmental toxicity of cefazolin sodium impurities in zebrafish. *Front. Pharmacol.* 8:403 10.3389/fphar.2017.00403 PMC5483477 28694779

[B8] Cherkasov A. Muratov E. N. Fourches D. Varnek A. Baskin I. I. Cronin M. (2014). QSAR modeling: where have you been? Where are you going to? *J. Med. Chem.* 57 4977–5010. 10.1021/jm4004285 24351051 PMC4074254

[B9] Edwards C. D. Luscombe C. Eddershaw P. Hessel E. M. (2016). Development of a novel quantitative structure-activity relationship model to accurately predict pulmonary absorption and replace routine use of the isolated perfused respiring rat lung model. *Pharm. Res.* 33 2604–2616. 10.1007/s11095-016-1983-4 27401409 PMC5040732

[B10] Fan Y. Shi L. Kohn K. Pommier Y. Weinstein J. (2001). Quantitative structure-antitumor activity relationships of camptothecin analogues: cluster analysis and genetic algorithm-based studies. *J. Med. Chem.* 44 3254–3263. 10.1021/jm0005151 11563924

[B11] Hwu J. R. Ethiraj K. S. Hakimelahi G. H. (2003). Biological activity of some monocyclic- and bicyclic beta-lactams with specified functional groups. *Mini Rev. Med. Chem.* 3 305–313. 10.2174/1389557033488132 12678824

[B12] Kislyuk S. Kroonen J. Adams E. Augustijns P. de Witte P. Cabooter D. (2017). Development of a sensitive and quantitative UHPLC-MS/MS method to study the whole-body uptake of pharmaceuticals in zebrafish. *Talanta* 174 780–788. 10.1016/j.talanta.2017.06.075 28738654

[B13] Li A. P. (2001). Screening for human ADME/Tox drug properties in drug discovery. *Drug Discov. Today* 6 357–366. 10.1016/S1359-6446(01)01712-3 11267922

[B14] Li A. P. (2004). Accurate prediction of human drug toxicity: a major challenge in drug development. *Chem. Biol. Interact.* 150 3–7. 10.1016/j.cbi.2004.09.008 15522257

[B15] Liu Y. (2018). Incorporation of absorption and metabolism into liver toxicity prediction for phytochemicals: a tiered in silico QSAR approach. *Food Chem. Toxicol.* 118 409–415. 10.1016/j.fct.2018.05.039 29782898

[B16] Liu Y. Zhang J. Hu C. (2018). Validated LC-MS/MS method for simultaneous analysis of 21 cephalosporins in zebrafish for a drug toxicity study. *Anal. Biochem.* 558 28–34. 10.1016/j.ab.2018.08.001 30081032

[B17] Lo Y. C. Rensi S. E. Torng W. Altman R. B. (2018). Machine learning in chemoinformatics and drug discovery. *Drug Discov. Today* 23 1538–1546. 10.1016/j.drudis.2018.05.010 29750902 PMC6078794

[B18] Macheboeuf P. Fischer D. S. Brown J. T. Zervosen A. Luxen A. Joris B. (2007). Structural and mechanistic basis of penicillin-binding protein inhibition by lactivicins. *Nat. Chem. Biol.* 3 565–569. 10.1038/nchembio.2007.21 17676039

[B19] Padilla S. Corum D. Padnos B. Hunter D. L. Beam A. Houck K. A. (2012). Zebrafish developmental screening of the ToxCast^TM^ Phase I chemical library. *Reprod. Toxicol.* 33 174–187. 10.1016/j.reprotox.2011.10.018 22182468

[B20] Pelka K. E. Henn K. Keck A. Sapel B. Braunbeck T. (2017). Size does matter - Determination of the critical molecular size for the uptake of chemicals across the chorion of zebrafish (*Danio rerio*) embryos. *Aquat. Toxicol.* 185 1–10. 10.1016/j.aquatox.2016.12.015 28142078

[B21] Qian J. Han Y. Li J. Zhang J. Hu C. (2018). Toxic effect prediction of cefatirizine amidine sodium and its impurities by structure-toxicity relationship of cephalosporins. *Toxicol. In Vitro* 46 137–147. 10.1016/j.tiv.2017.09.021 28963076

[B22] Raczakgutknecht J. Nasal A. Frąckowiak T. Kornicka A. Sączewski F. Wawrzyniak R. (2017). Comparative pharmacodynamic analysis of imidazoline compounds using rat model of ocular mydriasis with a test of quantitative structure-activity relationships. *J. Pharm. Biomed. Anal.* 144 122–128. 10.1016/j.jpba.2017.03.053 28420580

[B23] Rogers D. Hopfinger A. J. (1994). Application of genetic function approximation to quantitative structure-activity relationships and quantitative structure-property relationships. *J. Chem. Inf. Comput. Sci.* 34 854–866. 10.1021/ci00020a020

[B24] Shen J. Kromidas L. Schultz T. Bhatia S. (2014). An in silico skin absorption model for fragrance materials. *Food Chem. Toxicol.* 74 164–176. 10.1016/j.fct.2014.09.015 25290856

[B25] Singh G. S. (2004). Beta-lactams in the new millennium. Part-II: cephems, oxacephems, penams and sulbactam. *Mini Rev. Med. Chem.* 4 93–109. 10.2174/1389557043487547 14754446

[B26] Tal T. Kilty C. Smith A. LaLone C. Kennedy B. Tennant A. (2017). Screening for angiogenic inhibitors in zebrafish to evaluate a predictive model for developmental vascular toxicity. *Reprod. Toxicol.* 70 70–81. 10.1016/j.reprotox.2016.12.004 28007540 PMC6282194

[B27] Thareja S. (2015). Steroidal 5Î ± -reductase inhibitors: a comparative 3D-QSAR study review. *Chem. Rev.* 115 2883–2894. 10.1021/cr5005953 25785489

[B28] Wang T. Wu M. B. Lin J. P. Yang L. R. (2015). Quantitative structure-activity relationship: promising advances in drug discovery platforms. *Expert Opin. Drug Discov.* 10 1283–1300. 10.1517/17460441.2015.1083006 26358617

[B29] Zhang F. Qin W. Zhang J.-P. Hu C.-Q. (2015). Antibiotic toxicity and absorption in zebrafish using liquid chromatography-tandem mass spectrometry. *PLoS One* 10:e0124805 10.1371/journal.pone.0124805 PMC4418659 25938774

[B30] Zhang J. Meng J. Li Y. Hu C. (2010). Investigation of the toxic functional group of cephalosporins by zebrafish embryo toxicity test. *Arch. Pharm.* 343 553–560. 10.1002/ardp.201000005 20938949

[B31] Zhang J. Qian J. Tong J. Zhang D. Hu C. (2013). Toxic effects of cephalosporins with specific functional groups as indicated by zebrafish embryo toxicity testing. *Chem. Res. Toxicol.* 26 1168–1181. 10.1021/tx400089y 23848171

